# Differences in structural color and population genetic structure of Western and Central Palearctic *Polyommatus icarus* populations

**DOI:** 10.1038/s41598-026-59363-4

**Published:** 2026-07-01

**Authors:** Gábor Piszter, Lajos Szatmári, Gábor Sramkó, Krisztián Kertész, Levente Laczkó, Virág Krízsik, Zsolt Bálint, László Péter Biró

**Affiliations:** 1 Institute of Technical Physics and Materials Science, HUN-REN Centre for Energy Research, P.O. Box 49, H-1525 Budapest, Hungary; 2 HUN-REN–UD Conservation Biology Research Group, University of Debrecen, 1 Egyetem tér, H-4032 Debrecen, Hungary; 3 Juhász-Nagy Pál Doctoral School, University of Debrecen, 1 Egyetem tér, H-4032 Debrecen, Hungary; 4 Evolutionary Genomics Research Group, Department of Botany, University of Debrecen, 1 Egyetem tér, H-4032 Debrecen, Hungary; 5 Molecular Taxonomy Laboratory, Hungarian Natural History Museum, 13 Baross St, H-1088 Budapest, Hungary; 6 Department of Zoology, Hungarian Natural History Museum, 13 Baross St, H-1088 Budapest, Hungary

**Keywords:** Ecology, Ecology, Evolution, Genetics, Zoology

## Abstract

The blue structural coloration of male *Polyommatus icarus* butterflies functions as a sexual signaling trait and exhibits remarkable spectral stability within populations despite being generated by highly complex photonic nanoarchitectures. The correlation of the blue sexual signaling color and population genetic variation of the butterflies was investigated across the Western and Central Palearctic regions. Dorsal wing reflectance spectra was measured for 95 male specimens and compared with the population genetic structure revealed in 99 specimens by 18 recently developed microsatellites. Reflectance measurements indicated a clear separation between the European and Central Asian populations, consistent with our previous findings, while the intermediate populations near the Ural Mountains exhibited distinct European spectral characteristics. In contrast, genetic variation showed limited structuring and correlated primarily with geographic distance, as indicated by a significant isolation-by-distance pattern. Thus, although both reflectance and genetic variations are geographically structured, spectral properties are only weakly correlated with genetic differentiation. Populations near the Ural Mountains exhibited genetic ancestry linked to Central Palearctic groups, while displaying distinct Western Palearctic coloration, suggesting that the focal species’ sexual signaling is strongly influenced by local factors. These findings suggest that sexual signaling coloration may evolve at least partially independently of the neutral genetic background, offering additional insight into evolutionary divergence across broad geographic scales.

**Supplementary Information:**

The online version contains supplementary material available at https://doi.org/10.1038/s41598-026-59363-4.

## Introduction

Understanding how intraspecific phenotypic divergence emerges and persists among populations despite the homogenizing effects of both stabilizing selection and gene flow remains a central question in evolutionary biology. Although sexual signals are often subjected to strong stabilizing constraints to ensure successful mate recognition, they frequently exhibit substantial geographic variation across broader spatial scales. Structural coloration in butterfly wings provides a notable example of this pattern, where localized phenotypic variation may be maintained by multiple interacting evolutionary forces^1–3^. These include divergent ecological selection driven by thermoregulatory demands under varying regional climates^4^, reproductive character displacement in areas of sympatry with closely related taxa^5^, or simply historical neutral processes like genetic drift and isolation. Consequently, comparing phenotypic variation with neutral genetic structure is essential for determining whether geographic differences reflect adaptive responses to local selective pressures or instead result from demographic history and genetic drift. Building on previous findings, this study examines the relationship between macro-geographic variation in butterfly wing structural coloration and neutral evolutionary history inferred from microsatellites.

The focal species of our study, the Palearctic Common Blue butterfly, *Polyommatus icarus* (Rottemburg, 1775), is a well-suited model species to investigate the effects of continent-wide colonization and adaptation because of its abundance and widespread distribution^6,7^. The typical blue coloration of the dorsal wing surface of male *Polyommatus icarus*, from which its vernacular English name of the subtribe Polyommatina is even derived (Blues), is not generated by pigments; rather it is of structural origin^8^. Structural color is produced by photonic nanoarchitectures—also known as photonic band gap (PBG) materials^9^—which are nanocomposites composed of two transparent materials with sufficiently different refractive index to form a structure that prevents light of certain wavelengths from propagating through the photonic nanoarchitecture, causing it to be reflected instead. In butterflies, the two transparent materials are chitin and air^10^. The overall color observed on butterfly wings results from a mosaic of numerous wing cover scales, typically measuring 100 × 50 × 1 µm^3^, arranged regularly, like tiles on a roof^11^.

As with most phenotypic traits, variation in the structural coloration of *Polyommatus icarus* males can be observed in any given population. However, the extent of this variation is surprisingly small, especially when considering the complex process by which the reflected spectrum is generated. The natural spectral position of the reflectance maxima of male wings collected from a single population typically falls within a narrow a wavelength range of ±10 nm^12^. This may be attributed to the fact that these species are sexually dimorphic: the dorsal wing surfaces are brown for females and blue for males, with the latter color being produced by PBG materials. The blue color is used as sexual signaling color, which has a role in prezygotic communication^3^. As a result, males with an inappropriate color have fewer opportunities to transmit their genetic information to the next generation. This strong selective pressure leads to the conservation of male wing coloration and, consequently, of the underlying PBG materials. Alterations of just a few nanometers in the characteristic dimensions of the photonic nanoarchitectures produce a perceptible shift in the reflectance peak^11^^,^^13^. In other words, success in mate selection requires that the color generating photonic nanoarchitectures be reproduced with nanometer precision and should be inherited intact. As structural color is a sexual signal, the question arises as to whether a shift of more than 10 nm in the population-level average of the reflectance maxima constitutes an evolutionary signal.

A recent study has shown that males of *Polyommatus icarus* exhibit a characteristic difference in blue sexual signaling coloration between Europe and Asia^14^. Within Europe, there is good concordance between the peak wavelength of the reflectance maxima and the phylogeographic structure of four tested populations along a 1600 km longitudinal gradient as revealed by 10 recently developed DNA microsatellites^15^. The structural color was also temporally stable, as measurements of specimens collected from the same population over the past 100 years yielded consistent results^14^, demonstrating that both fresh and museum samples retained the same blue coloration. This remarkable structural and spectral reproducibility of the species-specific photonic nanoarchitectures can be utilized in potential applications^16^ or as templates of future artificial photonic materials^17^. Recently, we showed that laboratory breeding of this species can result in stable structural colors with lower variance than those observed in natural populations even though genetic erosion was detected after four generations of inbreeding^18^.

Each scale on the butterfly wing, along with the highly reproducible photonic nanoarchitecture it contains, is formed individually by scale-generating cells^19,20^. Despite several decades of investigation^21–25^, the precise mechanism by which the photonic nanoarchitectures—usually occurring in the lumen of the cover scales or on their modified ridges^23^—are produced with such a remarkable precision has not yet been fully elucidated.

Classical phylogeographic studies mostly rely on the 658 base pair (bp) long mitochondrial DNA barcoding region of the cytochrome c oxidase subunit I (COI) gene^26–30^. Recent work on our focal species *Polyommatus icarus* has reported a clear mitonuclear discordance on the British Isles, consistent with independent demographic histories of mitochondrial versus nuclear DNA^31^. Similar mitonuclear discordance was reported for the satyrid *Coenonympha oedippus* in the European Alps^32,33^. In the present work, we use previously developed nuclear DNA microsatellites—also called Single Sequence Repeats (SSRs)— for *P. icarus*^15^, which are useful for tracing genetic variability of natural populations due to their exceptionally high mutation rate (between 10^-6^ and 10^-2^ mutations per locus per generation^34^), and also facilitate comparison with our previous work^15^. Because *Wolbachia* infections have been shown to shape mitochondrial genetic structure in *Polyommatus icarus*^31^^,^^35^, and maternally inherited symbionts can confound phylogeographic inference from mtDNA through linkage with the mitochondrial genome^36^, neutral nuclear markers provide a more appropriate basis for reconstructing evolutionary history in this species.

In this study, we investigate the extent to which the spectral differences previously identified between the structural colors of European and Asian *Polyommatus icarus* males^14^ are correlated with microsatellite-based evolutionary history and geographic distance. In contrast to previous studies focusing on nondestructive measurements of museum collections^14^^,^^37^, we used fresh samples and employed a more precise, integrating sphere-based light collection spectrophotometric measurement^38^. The fresh samples—when available—also facilitated the production of high-quality DNA extractions.

## Results

### Reflectance spectrum analysis

**Fig. 1:**
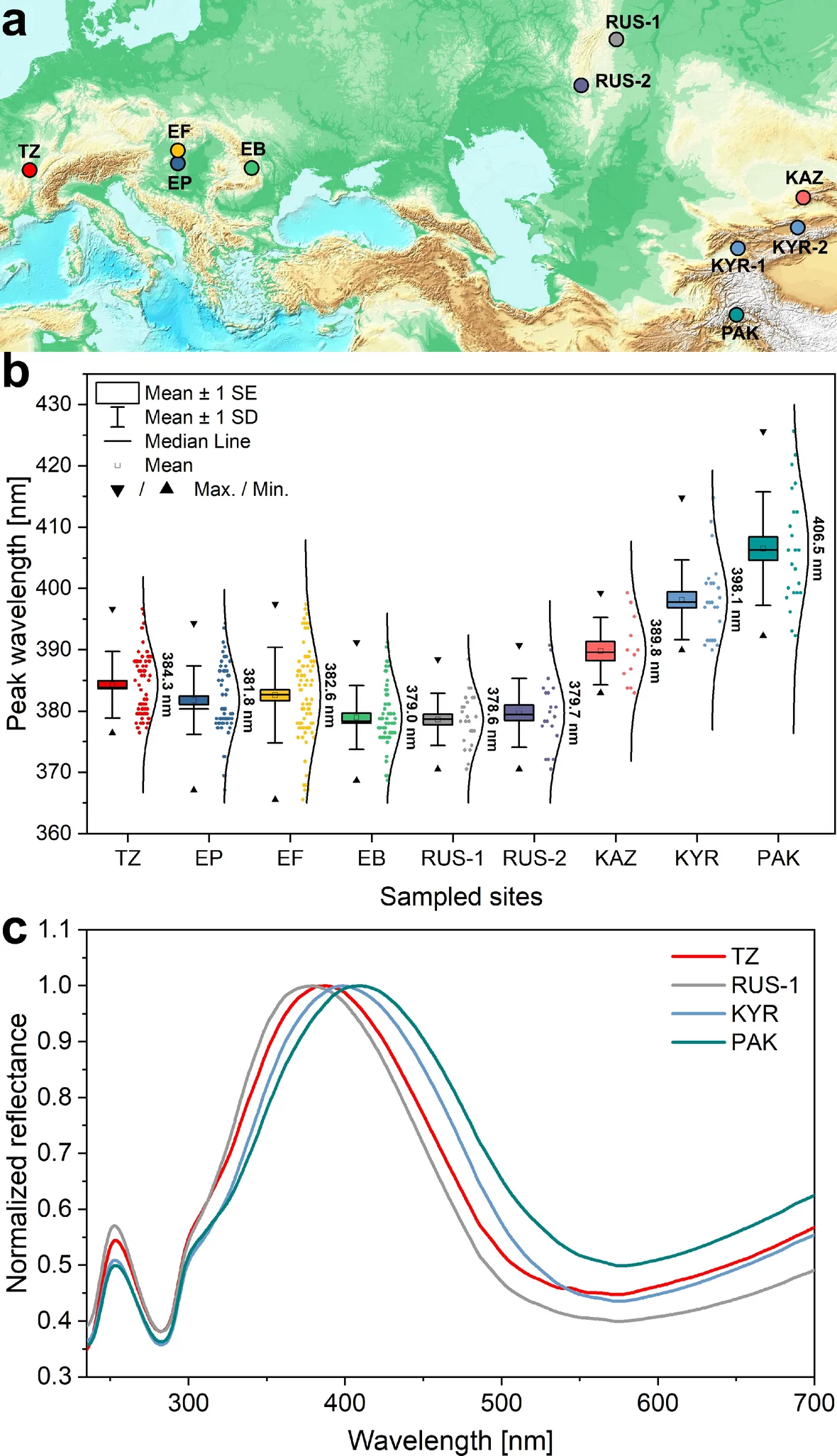
The measured peak wavelength values and calculated averages of the dorsal wing surface reflectance of male *Polyommatus icarus* specimens from the nine sampled populations from Europe and Asia. (**a**) The sampled locations are shown on the map. (**b**) Box diagrams, and (**c**) averaged reflectance spectra for all male specimens of the TZ, RUS-1, KYR, and PAK populations are presented normalized to their maximal reflectance amplitude.

The reflectance spectra were successfully measured for all available wing samples (Fig. 1). The calculated peak reflectance maxima distribution for all nine populations is shown in Fig. 1b. The range of individual values within populations rarely exceeded 20 nm. In contrast, between-population differences (average wavelength of reflectance maximum) were pronounced. For instance, in agreement with previous studies^14^, eastern populations (KYR, PAK) exhibited mean peak wavelengths approximately 20 nm higher than those of western populations. In line with previous findings based on different measurement techniques, the average reflectance maximum of all European populations fell within a 10 nm wavelength range^14,15^. The lowest average values were found in the Ural foothill populations (RUS-1, RUS-2 = 379 nm), closely matching the spectral characteristics of European populations, whereas the highest mean wavelength occurred in the southernmost population (PAK = 407 nm).

**Fig. 2:**
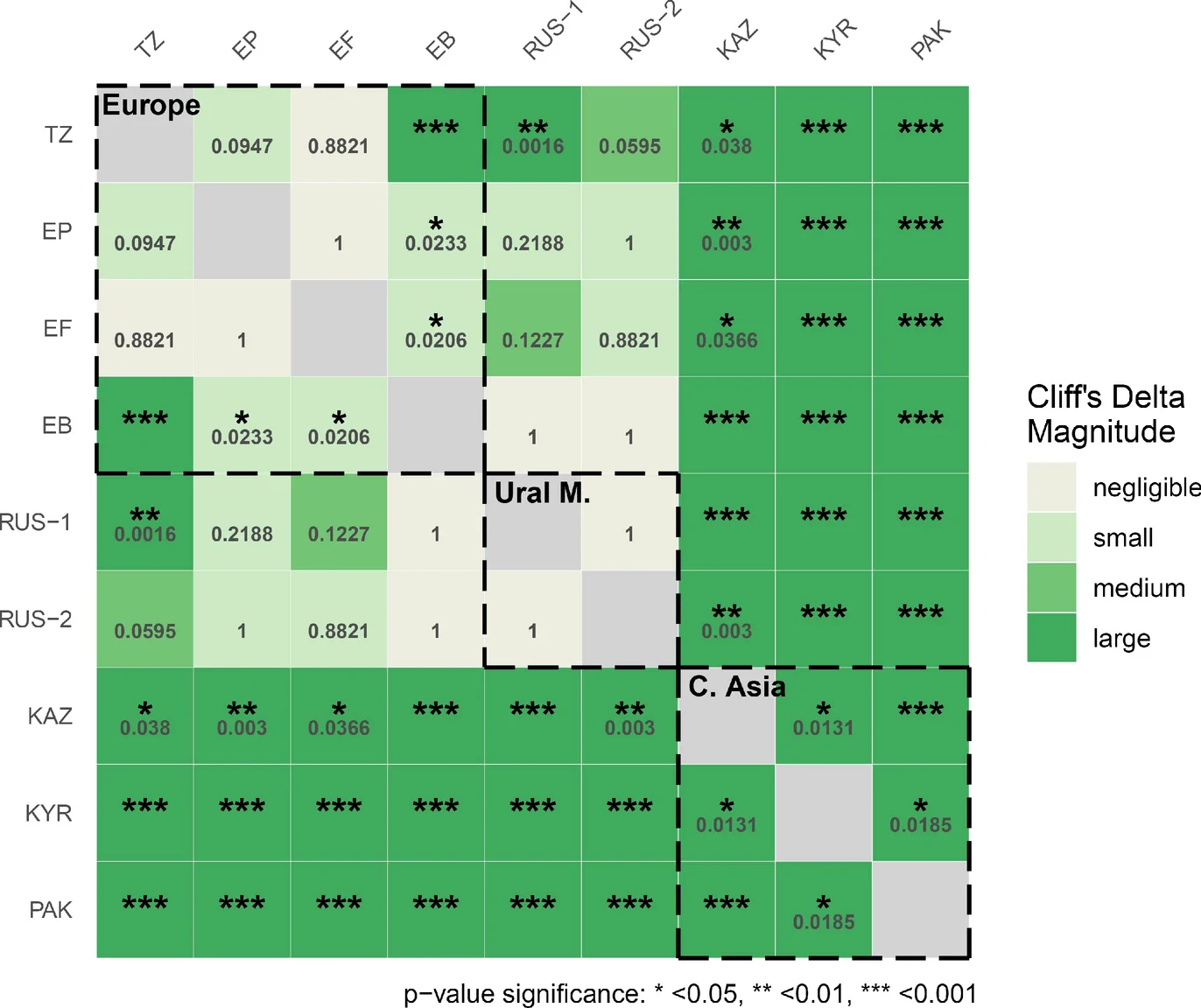
Heatmap of pairwise Cliff’s delta effect sizes for wing structural color among the studied populations, annotated with Wilcoxon rank-sum test p-values.

Peak reflectance wavelength differed significantly among populations (Kruskal–Wallis: χ^2^ = 156.5, df = 8, p < 0.001), indicating population-level variation in spectral phenotype. Post-hoc Wilcoxon rank-sum tests with Holm correction, supported by Dunn’s tests, showed that the largest differences occurred between Central Asian populations (KYR, PAK) and Central European populations (EB, EF, EP), with Central Asian populations exhibiting higher peak wavelengths (p < 0.001; large effect sizes; mean spectral shift ≈ 20 nm; Fig. 2). However, these tests indicate differences among population distributions rather than discrete spectral clusters. The overall pattern is therefore best interpreted as pronounced geographic variation in peak wavelength, with stronger differentiation involving the eastern populations and weaker differences among several European populations.

The sole exception within the European set was the Transylvanian population (EB), which differed significantly from the French population (TZ) with a large effect size. EB also differed significantly from the geographically proximate Hungarian populations (EF, EP), although the absolute differences in structural color were very small (Fig. 2), consistent with effect-size estimates indicating negligible divergence. Notably, EB had the lowest average reflectance maximum among the European groups (379 nm), nearly identical to the Ural foothill populations (RUS-1, RUS-2). Accordingly, pairwise comparisons between EB and the Ural populations were not significant, with effect sizes indicating negligible differences in wing color (Figs. 1 & 2).

The isolation-by-distance test at the individual level (Mantel test) revealed only a weak relationship between the geographic distance and the spectral differences, suggesting that geographic distance explains only a limited fraction of the observed spectral variation (Fig. S1a,b). The spectral properties of the populations were visualized via multivariate analysis based on the peak wavelength values of the measured reflectances. The UPGMA dendrogram based on peak wavelength differences summarized the same pattern of spectral dissimilarity: Central Asian populations, particularly KYR and PAK, were separated by larger spectral distances, whereas European and Russian populations showed smaller pairwise differences and tended to group together (Fig. S1c). Because this dendrogram is based on a single derived spectral-distance matrix, it should be interpreted descriptively rather than as evidence for discrete spectral clusters.

To directly compare spectral and genetic differentiation, we performed an additional Mantel test between pairwise peak-wavelength distances and pairwise genetic distances using the eight populations for which both datasets were available. This analysis revealed a moderate positive association, but the relationship was not statistically significant at the 0.05 level (Mantel test, r = 0.446, p = 0.0507, Fig. S2).

### Population genetic analysis

The initial check of nineteen microsatellite loci that were genotyped, only the PICA25 was monomorphic and therefore it was excluded from further analysis. As none of the remaining eighteen loci showed signs of significant deviation from Hardy-Weinberg equilibrium (HWe, Supplementary Table S2), no additional loci were excluded from the analysis. The resolution power of the final set of eighteen loci, as assessed using POWSIM, demonstrated reliably high performance (Supplementary Table S3). In all 100 cases of executed simulations, our microsatellite dataset provided sufficient information to significantly (p < 0.05) differentiate the populations at G_ST_ = 0.0245, as indicated by both the Chi-squared test and Fisher’s exact test. At the same time, the test for alpha error remained at an acceptably low level (Chi-squared test = 0.15 and Fisher’s exact test = 0.06). Nei’s overall genetic differentiation was found to be low (G’_ST(Nei)_ = 0.024).

Despite the high-resolution power of our microsatellite dataset, it did not recover biologically meaningful clustering. At the most probable value of K inferred by STRUCTURE (K = 2) (Fig. S3), mean individual assignment probabilities were approximately equal between the two inferred clusters, indicating no clear separation among individuals or sampling localities (Fig. S4). Inspection of additional STRUCTURE runs at higher K values likewise revealed no geographically coherent or interpretable substructure (Fig. S5): the extra clusters appeared as broadly shared ancestry components across individuals rather than as discrete groups corresponding to sampling sites or regions. Moreover, partitioning beyond K = 4 yielded technically uninterpretable results, with individual assignment probabilities dropping below 50% across all groups—a hallmark of spurious clusters^39^. Consistently, the assessment of the most probable number of clusters using sparse non-negative matrix factorization (sNMF), as implemented in LEA, supported the presence of a single ancestral population in the dataset (Fig. S6).

**Fig. 3:**
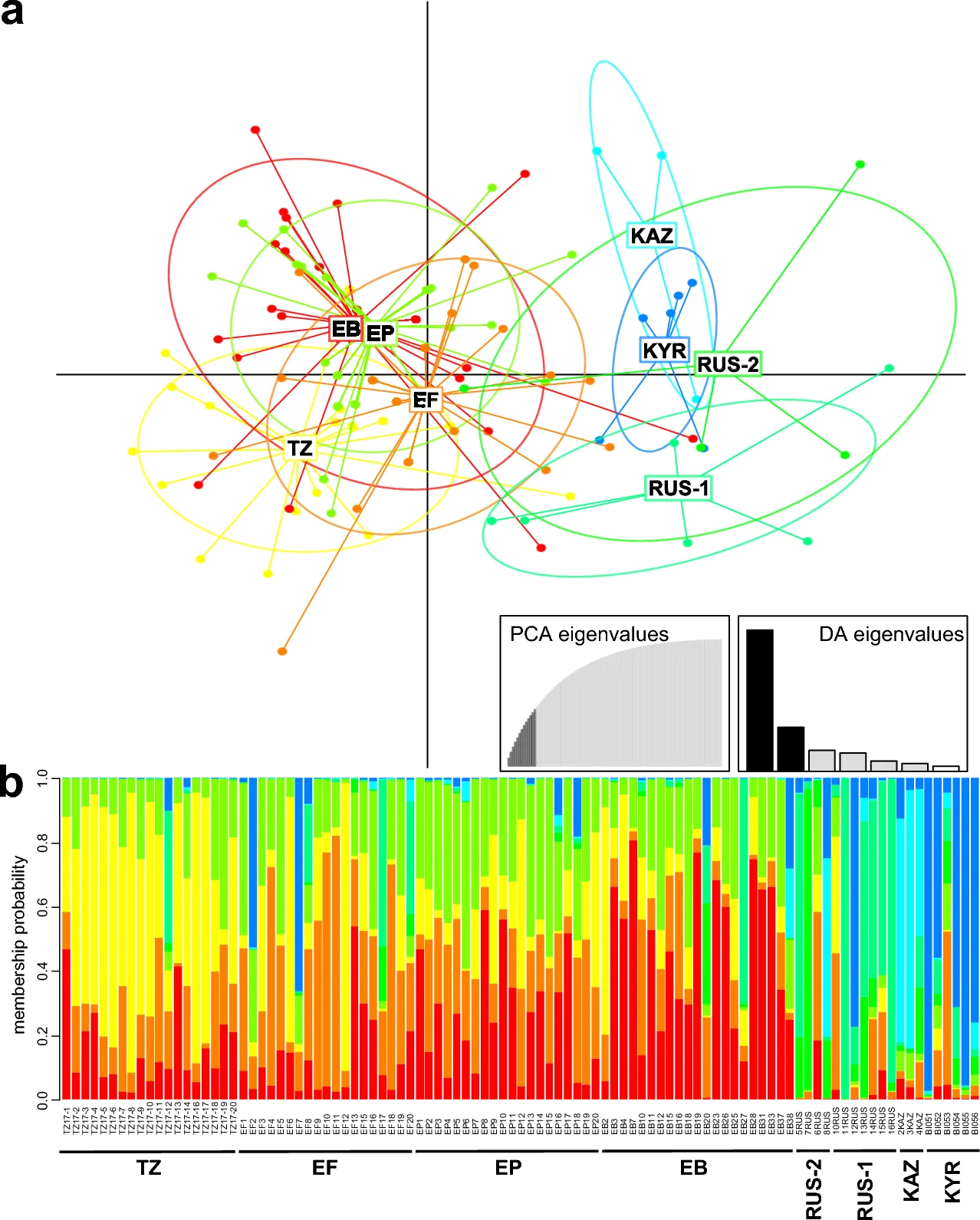
Individual-level discriminant analysis of principal components (DAPC) of the studied *Polyommatus icarus* individuals. (**a**) Largely overlapping inertia ellipses of the investigated populations are shown. Note the separation between Europe and Asia. (**b**) The membership proportions of individuals to the accepted original populations as a priori grouping show a rather admixed genetic background, although there is a slight west-to-east trend in what is the prevailing genetic compartment.

The individual-level PCA explained a mere 14.1% of the whole variance on the first three axes showing full mixture of the population at the individual level (Fig. S7). Therefore, we shifted our focus to another ordination approach, discriminant analysis of principal components (DAPC) analysis (Fig. 3), which maximized between-group variance by using information from 11 PCA axes and three axes in the linear discriminant analysis step as indicated by the DAPC cross-validation procedure. In the results of the DAPC, a mixing of individuals was also observed between the populations as indicated by the largely overlapping inertia ellipses of the groups (Fig. 3a). In accordance, the membership proportions of individuals show a rather admixed genetic background, although there is a slight west-to-east trend in what is the prevailing genetic compartment (Fig. 3b). This is reflected by the dominance of the genetic component of the French population (TZ) that is replaced by another such component in the Hungarian populations (EF, EP) and Romanian population (EB), which then gives way to the Russian (RUS-1, RUS-2) and Central Asian (KAZ, KYR) genotypes. Notably, different genotypes are dominant in the Asian samples compared to the European ones, but no clear clustering can be observed.

**Fig. 4:**
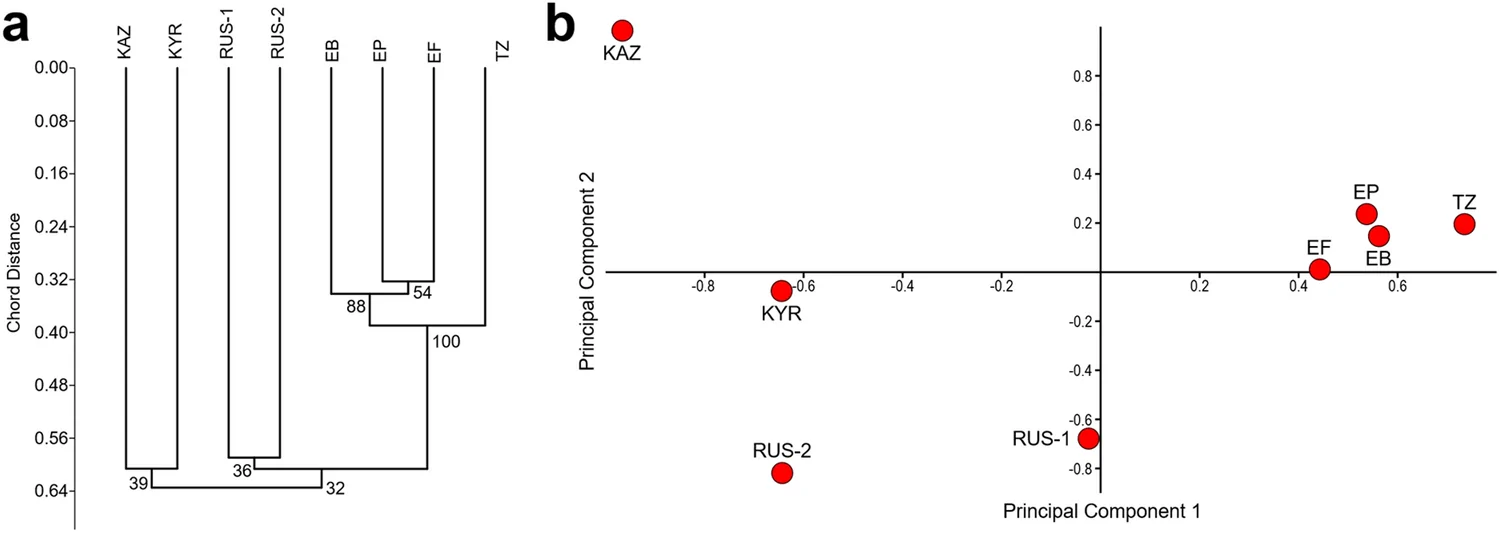
The genetic relationship between the populations is also visualized via multivariate analysis based on raw allelic frequencies. (**a**) The unweighted pair group method with arithmetic mean (UPGMA) dendrogram constructed using the 18 nuclear microsatellites of the 99 successfully sequenced *Polyommatus icarus* butterflies of the investigated populations. (**b**) Principal component analysis (PCA) ordination along the first two axes that covers 56.5% of the total variation of the groups shown in **a** is presented. The European and the Asian samples are well separated.

The genetic relationship between the populations is also visualized via multivariate analysis based on raw allelic frequencies (Fig. 4). The bootstrapped dendrogram (Fig. 4a), constructed based on Chord distance of raw allelic frequencies in UPGMA clustering, separated only two branches with full support (bs: 100%): one represented by the eastern (i.e., Central Asian and Ural Mountains) populations and the other by the western (i.e., all other European) populations. There is one more statistically supported grouping on the UPGMA dendrogram (Fig. 4a): EF & EP (Hungary) and EB (Romania) populations form a well-supported (bs: 88%) branch. Notably, the branch lengths are much longer in the eastern group than in the western, which indicates larger genetic distances between the populations.

Basically, a similar picture is seen on the PCA ordination of populations along the first two axes, which covers a large proportion of total genetic variation (56.5%) (Fig. 4b): the eastern and the western groups are separated along the second principal component, and populations of the eastern group are separated along the first principal component. There are much larger distances between the populations in the genetic space within the eastern group.

**Fig. 5:**
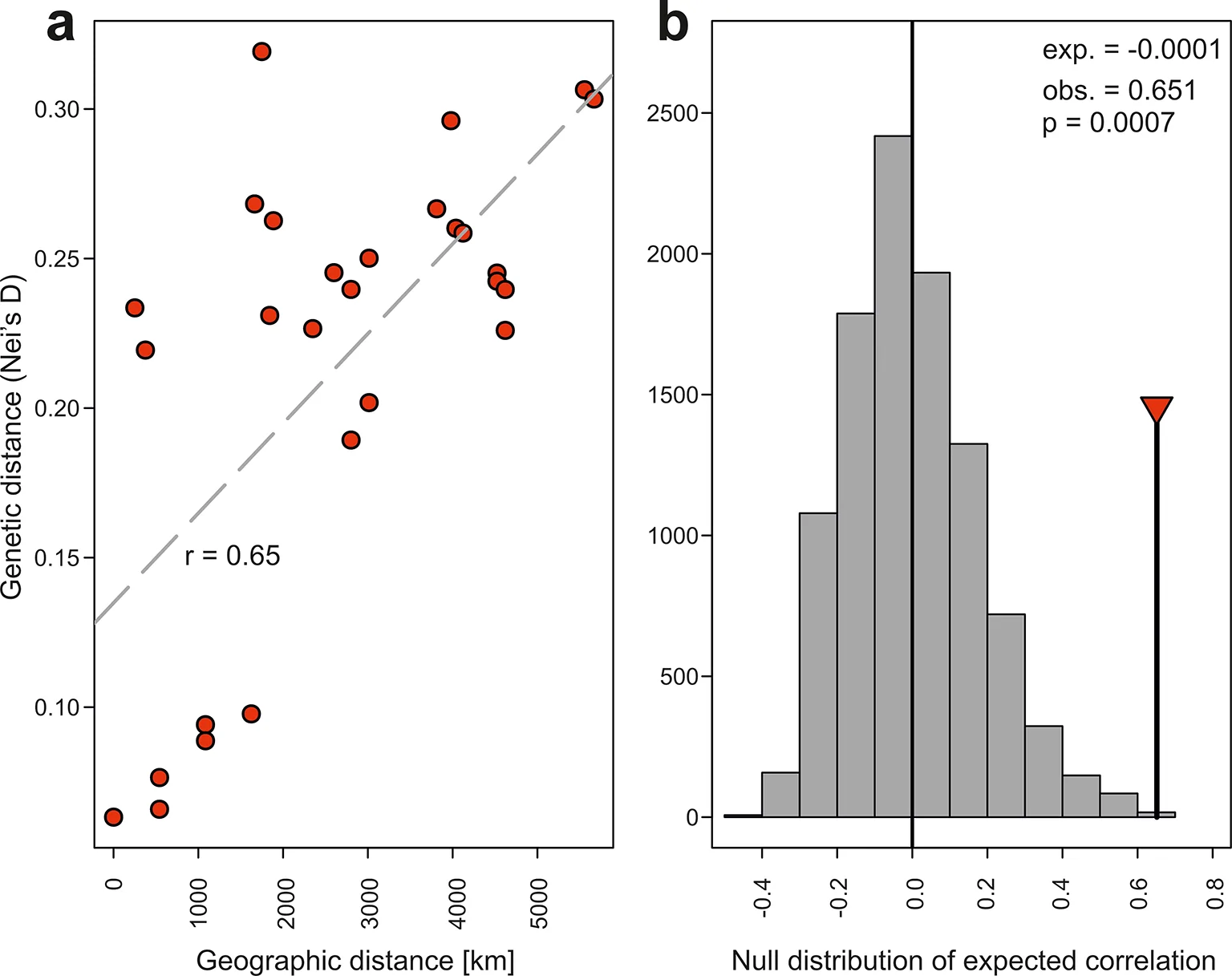
Test of isolation-by-distance at the population level (Mantel test). (**a**) Correlation between geographic distance and Nei’s genetic distance with the regression line. (**b**) Null distribution 10,000 simulated values of the null hypothesis compared to our observation (red triangle).

Finally, we have detected a significant isolation-by-distance as indicated by a strong positive correlation between geographic and genetic distances (Mantel test, r=0.651, p<0.01; Fig. 5). A similar but weaker pattern was observed between geographic (Fig. S8) and spectral distances, where the correlation was moderate but still significant (r = 0.367, p = 0.0192; Fig. S1a,b).

## Discussion

In essentially any species, genetic similarity among individuals is structured by the existence of subgroups and geographic isolation^40,41^. The membership assignment plot of the DAPC analysis (Fig. 3b) has become a *de* *facto* standard used as a non-parametric description of genetic data alongside PCA^41^. However, experienced researchers, particularly those interested in population structure and historical inference, stress the importance of presenting such results alongside other methods that make different modeling assumptions^42^. The use of precisely measured physical parameters, like structural color in the present work, may be a useful supplementary tool to achieve this. At the same time, genetic structuring can help in understanding the differences or similarities measured in structural coloration^14,15^. The new correlations revealed by the combined genetic-physical approach may complement the efforts to reveal the precise genetic mechanisms of color and pattern formation on butterfly wings^43–45^. Even for closely related species, the spectral differences of structural coloration are well correlated with the structural differences of the nanoarchitectures generating the species-specific structural colors^2,8^. Despite the clear mate selection role of color in butterfly species, significant structural color differences can be detected among different populations of the same species, even in such widely spread, habitat generalist species such as *Polyommatus icarus*^7,14^. Analysis of neutral genetic markers such as microsatellites can help understand the influence of evolutionary or phylogeographic history on the differences or similarities measured in structural coloration^15^. Indeed, there was a moderate positive association between spectral and genetic distances, but this relationship did not reach conventional statistical significance (Mantel test, r = 0.446, p = 0.0507, Fig. S2).

Analysis of the spectral characteristics of the investigated 95 male *Polyommatus icarus* specimens shows characteristic differences in structural color between European and Central Asian populations (Fig. 1). However, not all applied genetic analysis methods were able to identify this separation. The split between the two above-mentioned regions is well supported and clearly displayed on the UPGMA dendrogram (Fig. 4a). But, despite of the very high statistical power of our microsatellite dataset (see POWSIM analysis), the Bayesian clustering methods (Structure-analysis and LEA) failed to find any structure (Figs. S3–S6) within the studied sample group (Figs. 3 and 5). Most probably, these slightly controversial genetic results are simply the reflection of the autocorrelation between geographic and genetic distances clearly demonstrated by the significant results of the isolation-by-distance analysis (Fig. 5). From the point of view of male recognition, sexual signaling color within an expanding population, it may be disadvantageous to deviate from the characteristic color of the base population. The more so that at the frontier of the newly colonized region(s) the population density may be low, and the chance of successful mating encounters may be lower than in the base population. The shift towards the UV region in the spectral maximum of the European populations (Fig. 1b,c) may be associated with the lower altitude of the sampling sites, i.e., the weaker UV irradiation of the natural sunlight as compared with the Central Asian, high-altitude sampling sites. This pattern is most probably consistent with the trade-off between sexual signaling efficiency and predation risk: under lower UV conditions, males require more reflective structural coloration to remain visible to females, whereas in high-altitude environments with stronger UV irradiation, sufficient signaling efficiency can be achieved with less reflective coloration, thereby reducing conspicuousness to predators^46,47^. It is commonly believed that the *Polyommatus* genus originated from the high-altitude Himalayan region^48^.

Earlier, similar geographical distance-related differences were reported for the Palearctic region for the complex pattern on the ventral side of the wings of *P. icarus* butterflies^7^. In agreement with the data characterizing the structural coloration on the dorsal wing surface of the males, the pattern elements on the ventral wing surface define two large population groups: Western Palearctic and Central Palearctic^7^. This division concurs with a recent biogeographical investigation, which divided the Palearctic region into four sub-regions^49^. According to this division, our European samples belong to the European part of the Western Palearctic, while the Asian samples belong to Middle Asia (Central Palearctic).

Based on the measured reflectance spectra of the investigated specimens, from Western Europe to the Ural Mountains over a 4000 km range, only a minor variation in the average peak wavelengths is present in the reflectance maxima (5 nm). On the contrary, from the Ural Mountains to the Central Asian sites (KAZ, KYR, PAK populations) along distances of just 2000 km, the peak wavelengths are clearly shifted towards the red side of the visible spectrum (almost 20 nm). In other words, in Europe a lower spatial variation was found in the peak wavelength of the reflectance maximum when compared to Central Asia, where significant redshift and higher variations were measured. This variation in the structural color of males between the Western and Central Palearctic aligns well with the existence of two large population groups discussed earlier^14,15^. The border separating these two population groups is located between the foothills of the Ural Mountains and the Caspian Sea, in agreement with our previous work^14^, as well as with the findings of Artemyeva^7^ and the biogeographic division proposed by Kabalak and Sert^49^. Both the UPGMA dendrogram and the PCA data (Fig. 4) generated using genetic data support this division.

Wilcoxon rank-sum test results on spectral data confirm these findings (Fig. 2). Significant differences were found between the peak wavelength means of the European and Central Asian populations. The large Cliff’s delta (δ) effect sizes and the limited peak wavelength distributional overlap suggest that Central Asian individuals generally exhibit consistently higher peak wavelength values than their European counterparts. Moreover, these differences between population-level peak wavelengths far exceed the estimated spectral resolution of the butterfly visual system^50^, suggesting that the observed variation is likely perceptible to females. The Cliff’s delta (δ) effect size estimation, in combination with the rank-sum test, also reveals a much higher structural color diversity in Central Asian populations than in Europe.

The genetic differences visualized by the DAPC-based membership plot (Fig. 3b) indicated a largely admixed genetic background, although a weak west–east shift in the prevailing genetic component was apparent. The component dominant in the French population (TZ) was gradually replaced by another component in the Hungarian (EF, EP) and Romanian (EB) populations, which in turn gave way to components characteristic of the Russian Ural foothill (RUS-1, RUS-2) and Central Asian populations (KAZ, KYR). However, this neutral genetic pattern was not fully mirrored by spectral variation. In particular, the Ural populations showed genetic affinity towards the Central Asian populations, whereas their reflectance peak wavelengths were closer to those of European populations (Fig. 1, Fig. S1). Consistent with this partial mismatch, the association between spectral and genetic distances was moderate but did not reach conventional statistical significance (Mantel test, r = 0.446, p = 0.0507, Fig. S2).

The partial mismatch between neutral genetic structure and structural color variation is biologically informative. If spectral divergence were mainly the product of neutral drift following the same demographic history captured by microsatellites, a closer correspondence between genetic and spectral distances would be expected. Instead, the Ural populations were genetically closer to the Central Asian side of the west–east gradient but spectrally resembled European populations. Together with the non-significant Mantel correlation between spectral and genetic distances, this suggests that peak reflectance wavelength is not simply a by-product of neutral population structure. This pattern is compatible with a role for local selective pressures, environmental/developmental effects, or genotype–phenotype relationships not represented by the neutral marker set.

The genetic differences between Europe and Asia, and the lesser, but still significant differences of western European (TZ) and central and eastern European (EF, EP and EB) populations may be the consequence of the survival of thermophilous insects during glacial periods in refugia around the Mediterranean Sea^28^. As discussed by Dincă *et al.* in a continental-scale phylogeographical analysis of *Polyommatus icarus* in the Western Palearctic*,* the lineage termed ‘Palearctic’, appears to have expanded over large parts of southern Europe, too, and, possibly during glacial periods, it split into a southwestern European group (Ibero-Italian) and a central and eastern European one. Apparently, after the original colonization event, only a moderate gene flow from Asia to Europe occurred. A very similar, west-to-east colonization was recently reported for the wingless beetle *Lethrus apterus* (Geotrupidae, Coleoptera) and may indicate a more general pattern for grassland inhabiting insects^51^. Furthermore, the different environmental conditions of habitats can lead to directional selection of a certain trait^52^ that displaces the trait optimum towards one side of the distribution. In our case this varying trait is the structural color, the directional selection of which can first lead to increased variation, and then to the shift of the distribution to a new optimum. In this way, the driving force behind the changing structural color may be the presence of different species communities in habitats with varying climatic conditions, each offering distinct color environments to which the species have to adapt. Consequently, populations in the Ural Mountains, genetically Central Palearctic, may exhibit Western Palearctic structural coloration due to adaptation to European climate and habitat conditions (e.g., color composition of the local butterfly fauna^2,8^) for improved survival and reproductive success.

Understanding genetic and phenotypic divergence across populations is also important in elucidating ecological and evolutionary differences between populations. Such differences may be best observed when continent wide investigations are carried out^14^. Gradual genetic drift across populations (IBD pattern) and limited gene dispersal can account for some of the genetic and phenotypic divergence across populations. The combination of precisely measured physical characteristics with genetic data may help in identifying the initial stages of the divergence. Unfortunately, the EU Butterfly Indicator for Grassland species: 1990-2017^53^ shows a significant decline of butterflies, of 39%, during the investigated period. This decline raises the question: for how much longer will studies investigating on a continent-wide scale the correlation of well-measurable physical parameters of butterfly wing coloration with genetic characteristics will be possible in Europe?

## Conclusion

The structural coloration of male *Polyommatus icarus* shows clear geographic differentiation between Western and Central Palearctic populations, whereas neutral genetic variation is shaped mainly by isolation-by-distance and exhibits comparatively weaker population structuring. Although both datasets reveal broad west–east differentiation, the incomplete correspondence between spectral and genetic patterns, particularly in the Ural populations, suggests that structural coloration is influenced not only by demographic history but also by local selective pressures. Despite their genetic affinity to nearby Central Palearctic populations, the Ural populations display distinctly Western Palearctic spectral characteristics, closely resembling populations located nearly 3000 km farther west. This spectral shift may reflect adaptation to local environmental conditions, including differences in solar illumination and the habitat-specific color composition of the surrounding butterfly fauna. These findings indicate that sexual signaling coloration may evolve partly independently of neutral genetic background and can provide additional insight into evolutionary divergence across large geographic scales.

## Materials and methods

### Focal species

*Polyommatus icarus* (Rottemburg, 1775) (Lycaenidae: Polyommatinae: Polyommatini) is a widely distributed trans-Palearctic butterfly species. This species inhabits a range of open biotopes including chalk or limestone grasslands, woodland clearings, meadows, heathlands, sand dunes, secondary habitats along railway and road embankments, and abandoned urban sites over a range of elevational gradients from the Atlantic coast of Europe (including the British Isles) to the Pacific coast of Asia. Recently, the successful colonization of the Nearctic region by *P. icarus* has been reported^54,55^. The genus *Polyommatus* originated in Asia^48^, and the species *P. icarus* represents one of the youngest clades of the genus. The separation from the sister species is estimated to date about 1 million years ago^6^^,^^56,57^.

The number of annual broods varies between one and four, depending on climate, geography, and spatial factors. It is a generalist and an oligophagous species, with larvae primarily feeding on Fabaceae plants, including the widely cultivated *Lotus, Medicago*, and *Trifolium* species. *Polyommatus icarus* is not subject to any legal restrictions.

### Sampling

The detailed collection data of the European *P. icarus* specimens examined were previously published^15^. Extensive sampling of Central Asian *P. icarus* specimens was limited by the COVID-19 pandemic. However, in 2019, we were able to collect 15 males before the pandemic’s restrictions took effect. Additionally, 12 museum samples (6 from Kyrgyzstan and 6 from Pakistan) were included in the study. The museum samples are from 2007 and 1994 (see Supplementary Table S1). All specimens were destructively sampled: all four wings were removed for reflectance measurement purposes, and the bodies were used for DNA extraction. Neighboring collection sites represented by less than five samples were merged into one group for spectral and population genetic analyses. The nine sampling sites are shown in Fig. 1a and described in Supplementary Table S1.

### Reflectance spectroscopy

The wing reflectance was measured using an Avantes (Apeldoorn, The Netherlands) modular UV-visible spectroscopy setup consisting of a high sensitivity spectrophotometer (AvaSpec-HERO), a stabilized deuterium–halogen light source (AvaLight-DH-S-BAL), an integrating sphere (AvaSphere-30-REFL), and a diffuse PTFE reference tile (WS-2). The wavelength range of the reflectance measurements was 200–1000 nm. From the measured spectra, the peak wavelengths were calculated after 10 pts. FFT filtering using OriginPro 2021 (OriginLab Corporation, Northampton, MA, USA) software and were plotted in form of box plot diagrams. The reflectance spectrum was measured for each wing of all available male specimens (Fig. 1b, Supplementary Table S1). To facilitate the convenient comparison, the spectra were also normalized to their maximal amplitude.

### Reflectance spectrum analysis

From the broad range of the measured reflectance spectrum (200–1000nm), we focused on the 350–450nm range, as type I and type II ommatidium of the butterfly eyes contain photoreceptors (R1, R2) that are highly sensitive at the 350–450nm range^50^^,^^58^, granting the ability to distinguishing wavelength difference even as small as 1-2 nm. In addition, the focal species male wing reflectance spectrum not only resembles the spectrum of a monochrome surface color with one clear relatively narrow peak (Fig. 1c), but this peak also falls within 350–450 nm range (Fig. 1b). Furthermore, the reflectance profiles showed highly consistent shapes among individuals, differing primarily in the wavelength of maximum reflectance. Accordingly, peak reflectance wavelength was used as a single quantitative descriptor of wing color.

Differences in peak reflectance wavelength among populations were tested using non-parametric approaches. Normality was evaluated with the Shapiro–Wilk test and homogeneity of variances with Levene’s test, both of which indicated violations of parametric assumptions. We therefore applied a Kruskal–Wallis test to assess overall population differences, followed by pairwise comparisons using the Wilcoxon rank-sum test with Holm correction. Robustness of results was confirmed with Dunn’s test. All analyses were performed in R^59^.

Effect sizes were quantified using Cliff’s delta (δ), a non-parametric measure of stochastic dominance. This robust estimator quantifies the probability that a randomly selected observation from one group is larger than a randomly selected observation from the other, providing a measure of distributional overlap that remains valid under conditions of non-normality and heteroscedasticity. Conventional thresholds interpret |δ| < 0.147 as negligible, 0.147–0.33 as small, 0.33–0.474 as medium, and ≥ 0.474 as large^60,61^.

### DNA extraction

DNA extraction was carried out following the protocol outlined in reference^15^. Unfortunately, the six butterflies captured at the Shandur Pass in 1994 exhibited higher degrees of DNA degradation, which prohibited the amplification of PCR products. Therefore, we could not integrate these samples into the molecular genetic dataset.

### Population genetics

For the population genetic analysis, we employed a microsatellite marker set, which was developed and evaluated in a recent study^15^. Here we increased statistical power by expanding the number of microsatellite loci from 10 (used in the previous publication^15^) to 19. PCR amplification, microsatellite fragment length analysis, and genotyping methods also followed the procedures outlined in the aforementioned article. Raw microsatellite genotyping data of 19 loci were imported into MS Excel (Microsoft, Redmond, WA, USA) and analyzed using GenAlEx v.6.551^62^. One locus (PICA25) had to be excluded from all downstream analyses as it displayed no variation (Table S2). We first assessed deviations for each locus in each population from the Hardy–Weinberg-equilibrium (HWe). With the help of GenAlEx, we calculated Nei’s overall genetic differentiation between the populations (G’_ST(Nei)_)^63^, and this value was used as a target value in a simulation exercise using POWSIM^64^ based on actual allelic frequencies to test for the statistical power of our 18 loci in providing resolution if we simulate 100× a hypothetical ancestral population of 1000 individual to achieve the actual level of genetic differentiation between our studied populations. The GenAlEx datafile was imported into R by *poppr* v. 2.9.2^65^ and adegenet v.2.1.2^66^ was used to accomplish a principal component analysis (PCA) based on individual allele frequencies.

Two Bayesian clustering methods were used to assign individuals to genetic clusters: (1) the individual assignment and *k*-means clustering as implemented in Structure v.2.3.4^40^ and (2) sparse non-negative matrix factorization (snmf) from the R package LEA v.2.0^67^ that estimates ancestry coefficients and uses a cross-entropy criterion to assess ancestral population number (i.e., the best K value based on the data) using default settings. Structure analysis was run for 0.5 million generations after 100k ‘burn-in’ five times for each K from 1 to 8 using the R function ParallelStructure v.1.0^68^. The result of the run was evaluated using the on-line tool StructureHarvester v.0.6.94^69^ and considering deltaK as the indicator of the best grouping as suggested by Evanno et al^70^.

Given the limited variability depicted by PCA, we implemented a discriminant analysis of principal components (DAPC)^71^ that maximizes the discrimination between groups and information content of several PCA axes. As Bayesian clustering showed the lack of solid genetic structure in our data, we did not test the optimal number of groups by *k*-means of clustering in DAPC but accepted the original populations as *a priori* grouping. Thus, we only used DAPC to describe the variability of the genetic make-up of our studied individuals.

Population-level raw allelic frequencies from GenAlEx were imported into PAST v.2.17c^72^ to visualize genetic relationship between the studied populations by means of multivariate statistics using PCA and unweighted pair group method with arithmetic mean (UPGMA) tree building. The latter was constructed by first calculating Cavalli-Sforza chord distance then using the paired-group algorithm to build the dendrogram. The statistical test of branch robustness was tested by 1000 non-parametric bootstrap (bs). Finally, the statistical relationship between geographic distance and genetic distance at the individual level, and also the relationship between geographic distance and the position of the reflectance maxima wavelength (i.e., the test of isolation-by-distance, IBD) was analyzed via a Mantel test as implemented in ade4 v. 1.7-17^73^ based on the Bruvo distance^74^ by comparing the pairwise genetic, spectral and geographic distance of each individual in 10k permutations.

## Supplementary Information

Supplementary Information 1.

Supplementary Information 2.

## Data Availability

All relevant data analyzed during this study are included in this published article and its supplementary information files. Raw datasets used during the current study are available from the corresponding author upon reasonable requests.
