# Supplementary material for: Differences in structural color and population genetic structure of Western and Central Palearctic Polyommatus icarus populations
Source: Sci Rep. 2026 Jul 1;16:27661. doi: 10.1038/s41598-026-59363-4 (PMC13542078; doi:10.1038/s41598-026-59363-4)
Supplement: Supplementary file 1 [file 41598_2026_59363_MOESM1_ESM.pdf]

Supplementary Information

Differences in structural color and population genetic structure of Western and Central Palearctic *Polyommatus icarus* populations

Gábor Piszter, Lajos Szatmári, Gábor Sramkó, Krisztián Kertész, Levente Laczkó, Virág Krízsik, Zsolt Bálint, and László Péter Biró

Table S1. Data of the 106 *Polyommatus icarus* specimens used in the present study: sample name, sex, location and data of collection.

See Excel file *41598_2026_59363_MOESM2_ESM.xlsx*

Table S2. Deviations for each locus in each population from the Hardy–Weinberg-equilibrium (HWe) as calculated in GenAlEx v.6.551, and missingness at the population level (in the last row) plus at the locus level (in the last column).

		Baratcos (EB)	Érd-1 (EP)	Érd-2 (EF)	Teizé (TZ)	Orenburg (RUS-2)	Chelyabinsk (RUS-1)	Tekeli (KAZ)	Jalal-Abad & Yssyk-Kul (KIR)	missing data
	**N**	21	19	19	20	4	7	3	6	
**no.**	**Locus**									
1	PICA16	ns	ns	*	ns	ns	ns	ns	ns	0.0%
2	PICA12	*	ns	ns	ns	ns	ns	Monomorphic	ns	1.0%
3	PICA2	ns	ns	ns	ns	ns	ns	ns	ns	6.1%
4	PICA6	ns	ns	ns	ns	ns	ns	ns	ns	2.0%
5	PICA45	ns	*	*	ns	ns	ns	ns	ns	0.0%
6	PICA1	ns	ns	ns	**	ns	ns	ns	ns	1.0%
7	PICA25	Monomorphic	Monomorphic	Monomorphic	Monomorphic	Monomorphic	Monomorphic	Monomorphic	Monomorphic	1.0%
8	PICA23	ns	ns	**	ns	ns	ns	ns	ns	0.0%
9	PICA24	Monomorphic	ns	ns	***	Monomorphic	Monomorphic	Monomorphic	ns	3.0%
10	PICA39	ns	ns	ns	ns	ns	ns	ns	ns	1.0%
11	PICA22	*	ns	**	ns	ns	**	ns	*	12.1%
12	PICA10	ns	ns	ns	ns	ns	ns	ns	**	4.0%
13	PICA27	ns	ns	ns	*	ns	ns	ns	ns	2.0%
14	PICA37	***	*	**	**	ns	ns	ns	ns	0.0%
15	PICA36	ns	**	ns	ns	ns	ns	ns	ns	5.1%
16	PICA42	ns	ns	ns	ns	Monomorphic	ns	ns	ns	1.0%
17	PICA21	ns	ns	*	ns	Monomorphic	ns	Monomorphic	ns	1.0%
18	PICA19	ns	ns	ns	**	ns	ns	ns	ns	4.0%
19	PICA34	ns	ns	*	ns	ns	ns	ns	*	3.0%
missing data		1.3%	1.9%	2.2%	3.9%	0.0%	3.8%	3.5%	4.8%	

Table S3. Outcome of powersim analyzes. (**a**) analysis of actual data aiming at achieving the same level of differentiation between the samples after genetic drift; (**b**) analysis of alpha error without leaving to segregate populations.

(**a**) Proportion of significances (P<0.05) for summed/combined test statistics:							(**b**) Proportion of significances (P<0.05) for summed/combined test statistics:						
Loci	Alleles	Runs	#Chi2	Chi2	#Fisher	Fisher	Loci	Alleles	Runs	#Chi2	Chi2	#Fisher	Fisher
18	=<220	100	100	1.0000	100	1.0000	18	=<220	100	15	0.1500	6	0.0600
Tot	***	100	100	1.0000	100	1.0000	Tot	***	100	15	0.1500	6	0.0600
Average F'st over all the 100 runs = 0.0245							Average F'st over all the 100 runs = 0.0000						
Expected Fst = 0.0247							Expected Fst = 0.0000						
F'st calculated from average Hs and Ht = 0.0245							F'st calculated from average Hs and Ht = 0.0000						

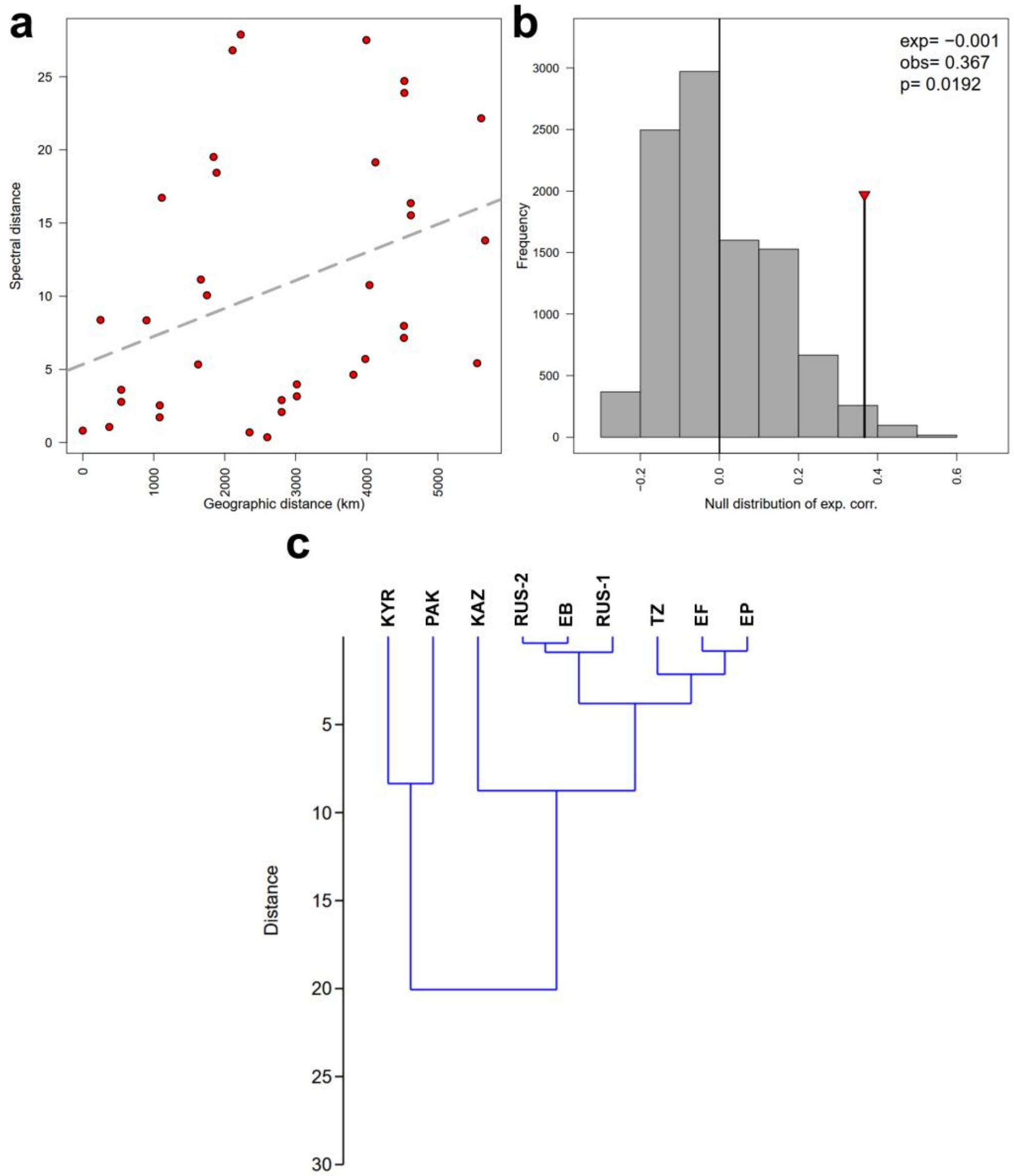

Fig. S1. Test of isolation-by-distance at the population level (Mantel test). (**a**) Correlation test between geographic distance and spectral distance with the regression line. (**b**) Null distribution 10,000 simulated values of the null hypothesis compared to our observation (red triangle). (**c**) The unweighted pair group method with arithmetic mean (UPGMA) dendrogram constructed using the peak wavelength data of the measured *Polyommatus icarus* butterflies of the investigated populations.

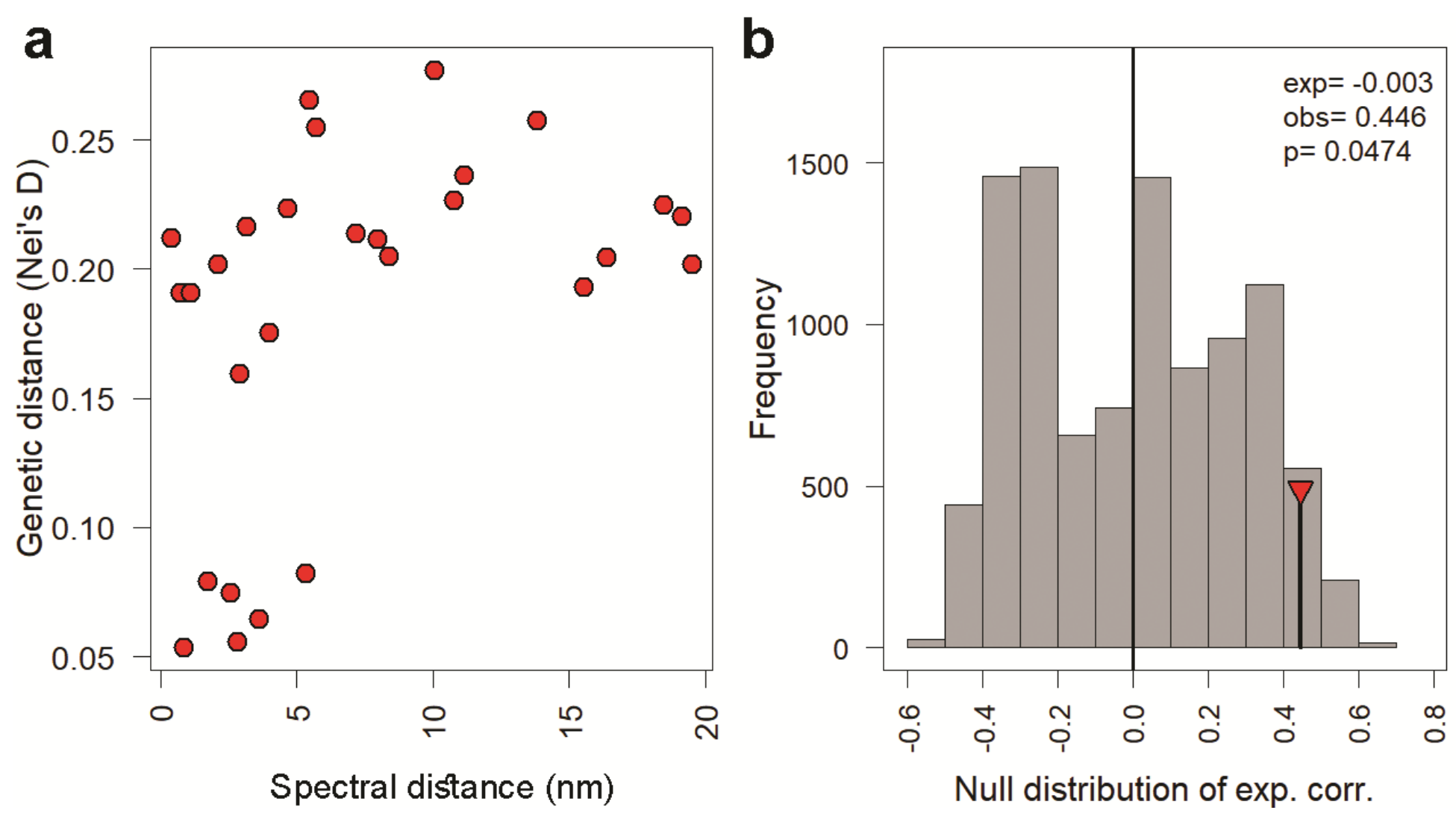

Fig. S2. Test of isolation-by-distance at the population level (Mantel test). (**a**) Correlation test between genetic distance and spectral distance with the regression line. (**b**) Null distribution 10,000 simulated values of the null hypothesis compared to our observation (red triangle).

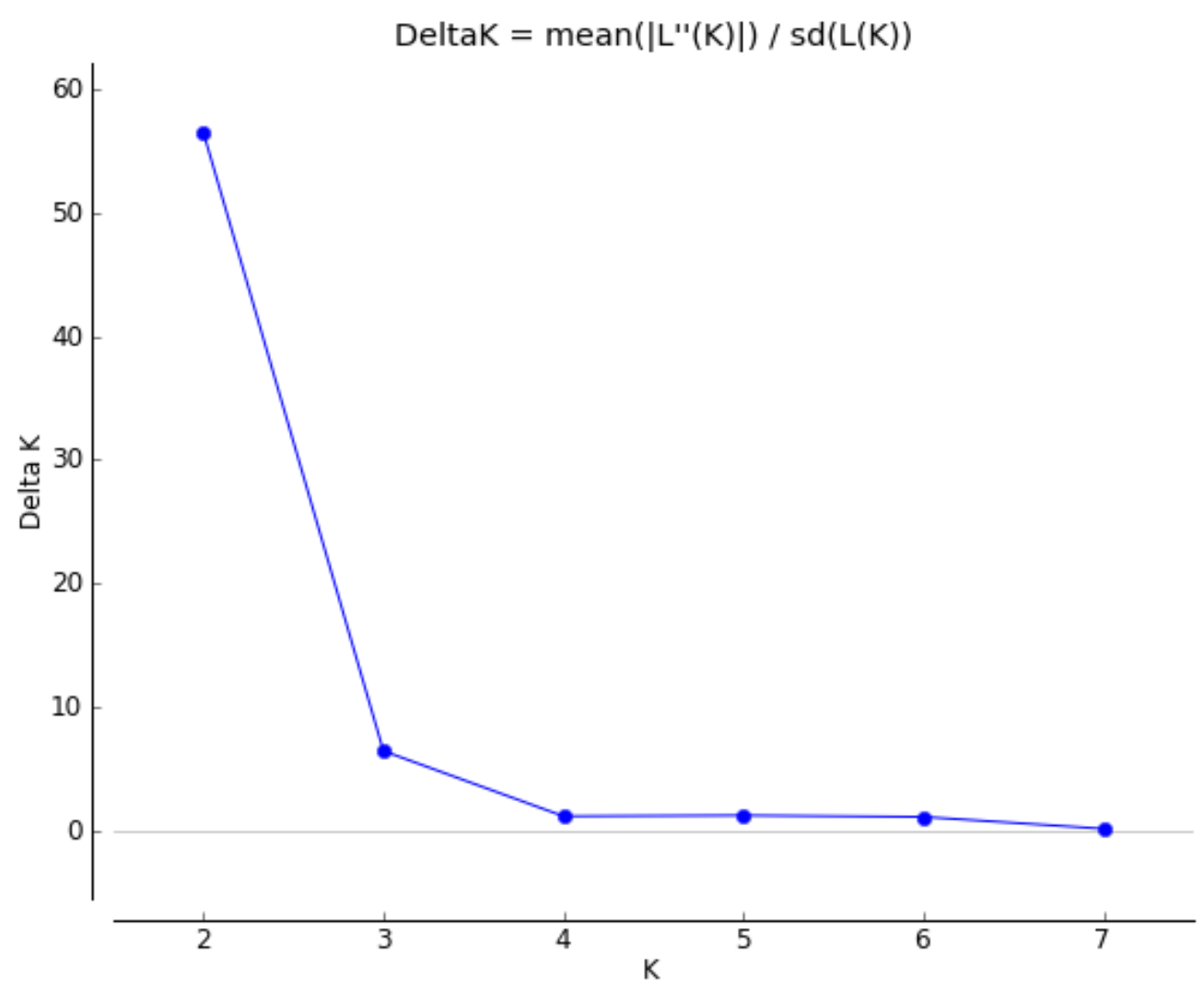

Fig. S3. Plot of deltaK exported from StructureHarvester v.0.6.94 based on Bayesian clustering of SSR data of 99 *Polyommatus icarus* individuals using the software Structure v.2.3.4.

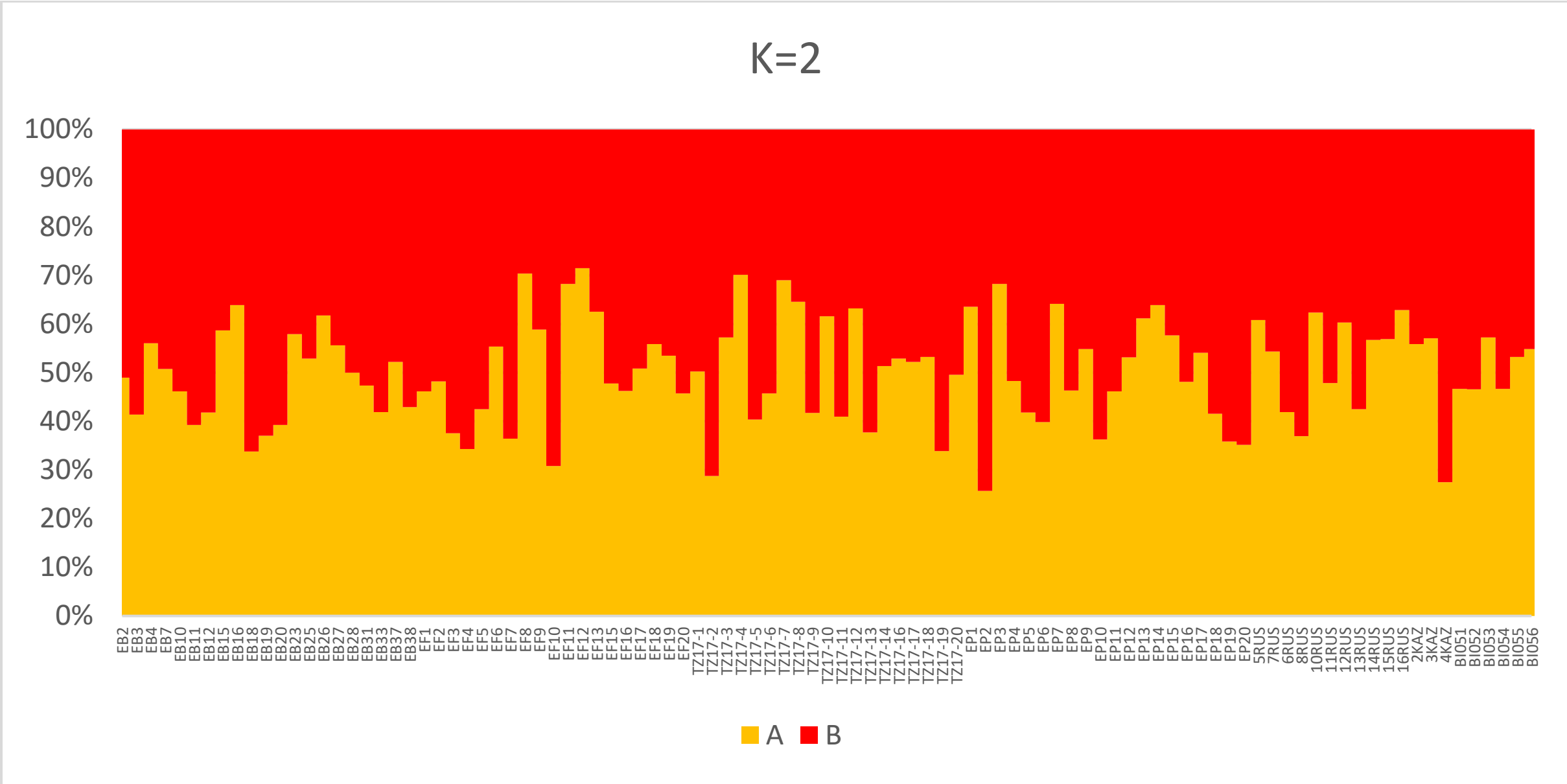

Fig. S4. Barplot of Structure analysis at the most probable K=2 as suggested by the method of Evanno et al. (2005).

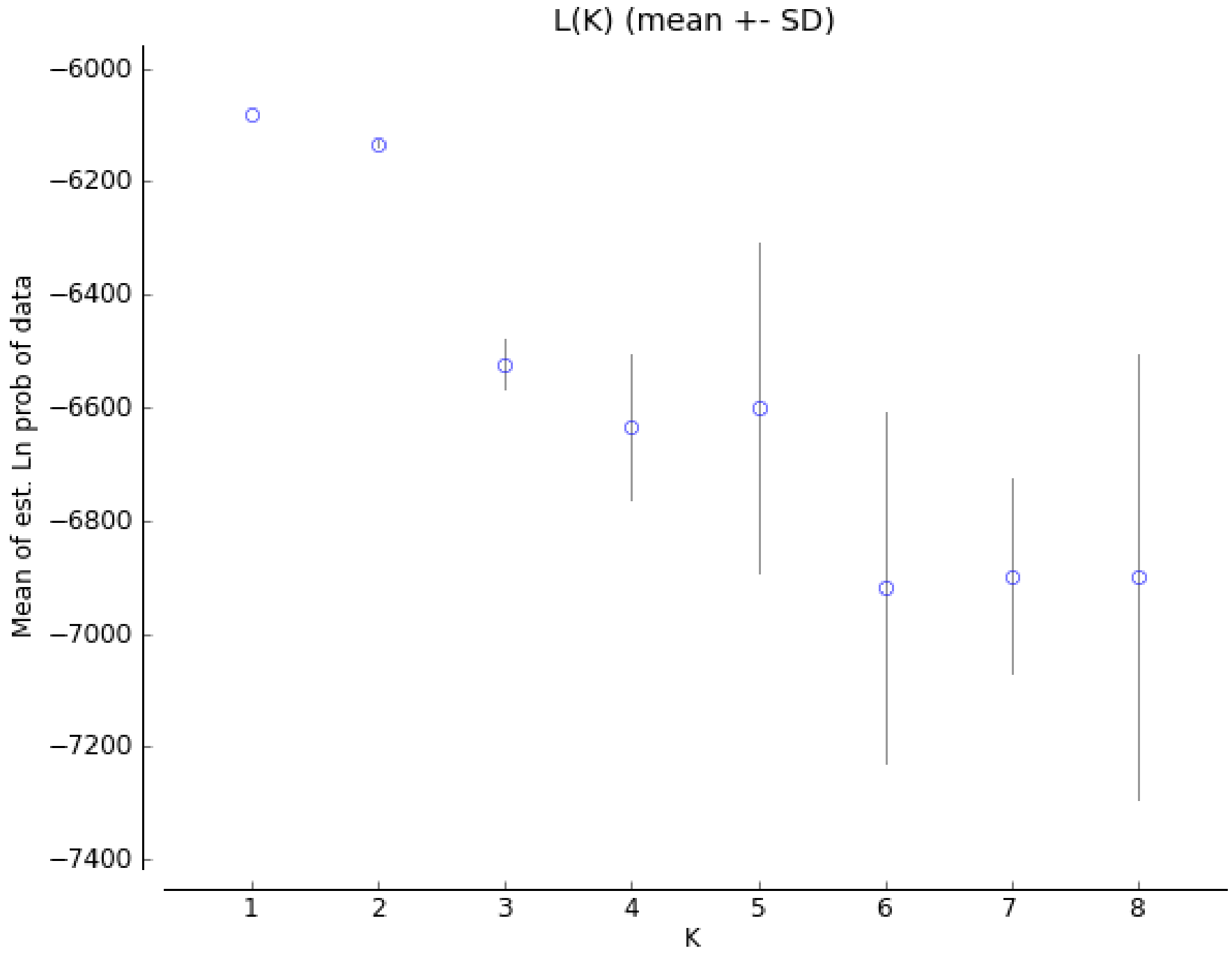

Fig. S5. Mean Ln Pr(X|K) values and their standard deviation as output from StructureHarvester v.0.6.94.

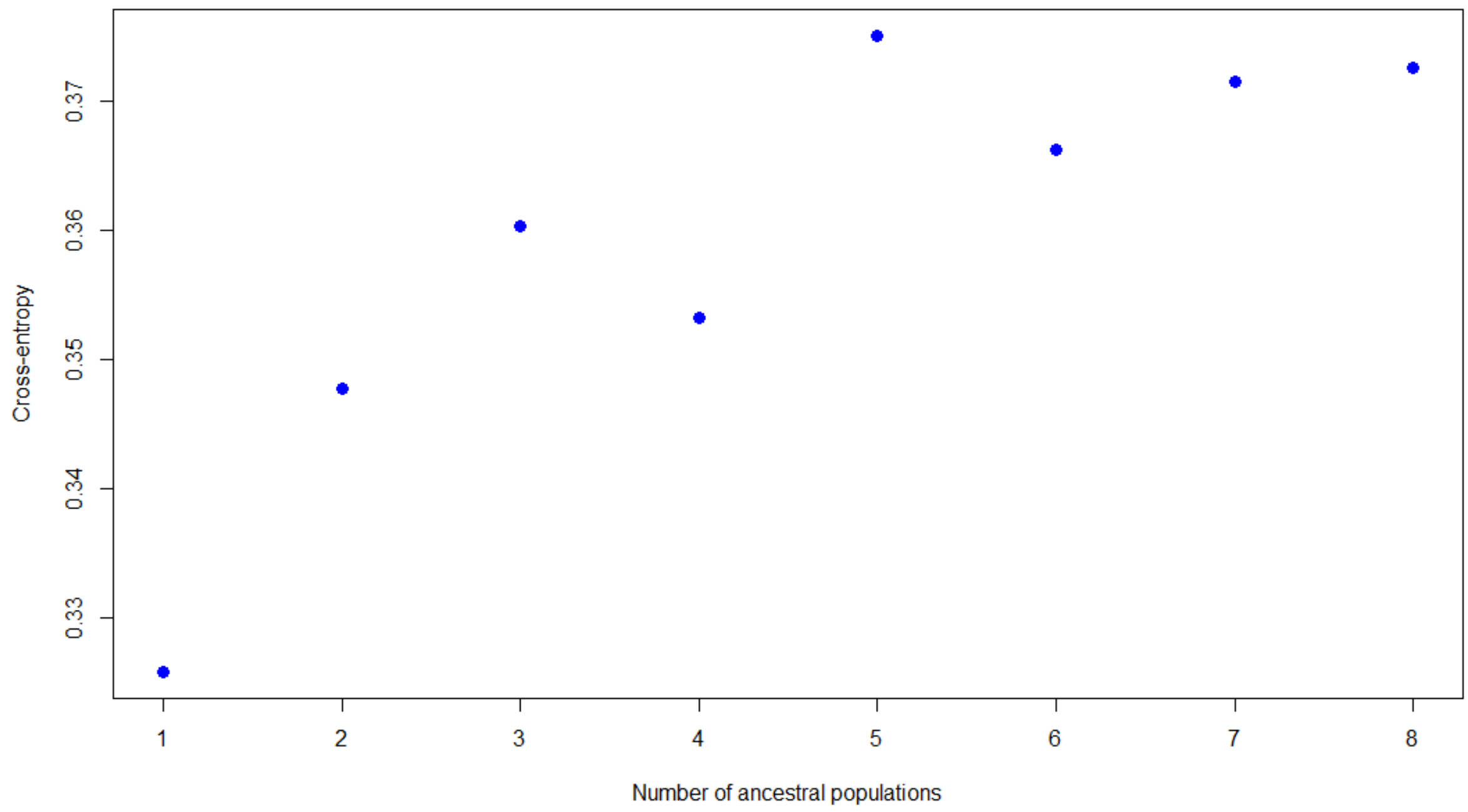

Fig. S6. Analysis of the number of genetic clusters based on cross-entropy criterion as estimated from sparse non-negative matrix factorization implemented in the package LEA v.2.0.

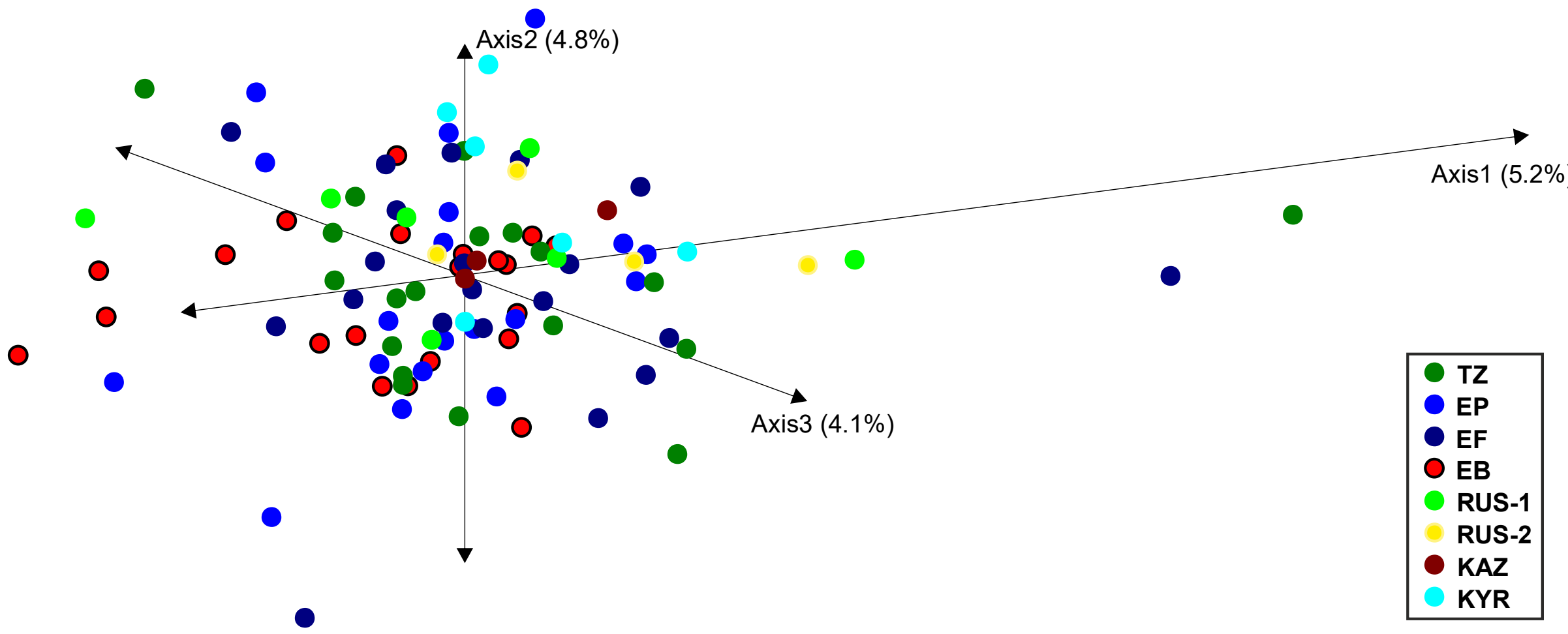

Fig. S7. Principal Component Analysis (PCA) plot of raw allelic frequency proportions at the individual level for the analyzed populations.

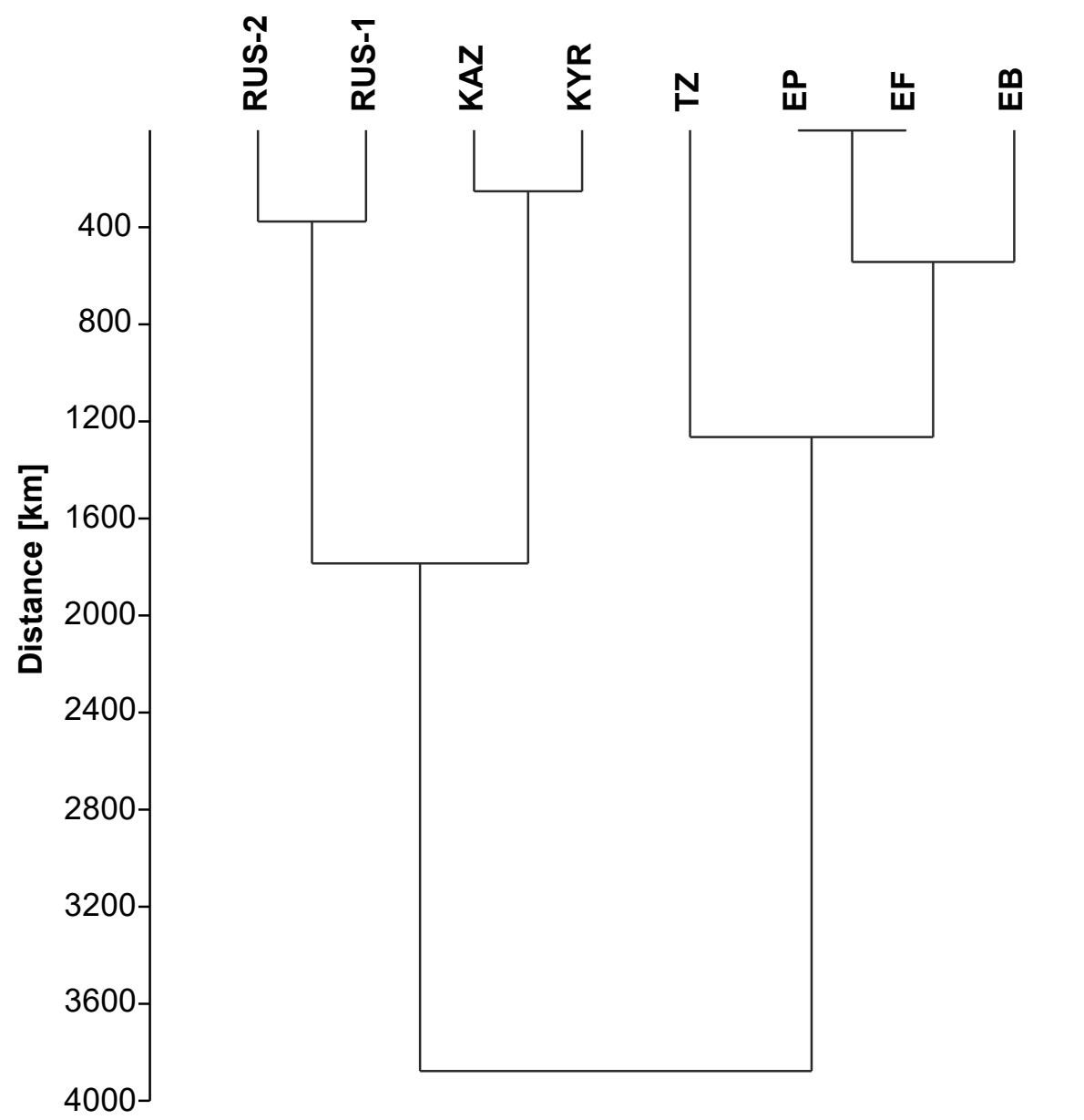

Fig. S8. Unweighted pair group method with arithmetic means (UPGMA) of geographic distances between the sampling locations.
